# Sulfated Polysaccharides from Marine Algae as a Basis of Modern Biotechnologies for Creating Wound Dressings: Current Achievements and Future Prospects

**DOI:** 10.3390/biomedicines8090301

**Published:** 2020-08-22

**Authors:** Boris G. Andryukov, Natalya N. Besednova, Tatyana A. Kuznetsova, Tatyana S. Zaporozhets, Svetlana P. Ermakova, Tatyana N. Zvyagintseva, Ekaterina A. Chingizova, Anna K. Gazha, Tatyana P. Smolina

**Affiliations:** 1 Somov Research Institute of Epidemiology and Microbiology, 690087 Vladivostok, Russian; besednoff_lev@mail.ru (N.N.B.); takuznets@mail.ru (T.A.K.); niiem_vl@mail.ru (T.S.Z.); angazha@mail.ru (A.K.G.); tsmol@mail.ru (T.P.S.); 2 School of Biomedicine, Far Eastern Federal University (FEFU), 690091 Vladivostok, Russian; 3 Elyakov Pacific Institute of Bioorganic Chemistry (PIBOC) FEB RAS, 690022 Vladivostok, Russian; svetlana_ermakova@hotmail.com (S.P.E.); zvyag@piboc.dvo.ru (T.N.Z.); martyyas@mail.ru (E.A.C.)

**Keywords:** seaweed, sulfated polysaccharides, alginates, fucoidans, carrageenans, ulvans, wound dressing, wounds

## Abstract

Wound healing involves a complex cascade of cellular, molecular, and biochemical responses and signaling processes. It consists of successive interrelated phases, the duration of which depends on a multitude of factors. Wound treatment is a major healthcare issue that can be resolved by the development of effective and affordable wound dressings based on natural materials and biologically active substances. The proper use of modern wound dressings can significantly accelerate wound healing with minimum scar mark. Sulfated polysaccharides from seaweeds, with their unique structures and biological properties, as well as with a high potential to be used in various wound treatment methods, now undoubtedly play a major role in innovative biotechnologies of modern natural interactive dressings. These natural biopolymers are a novel and promising biologically active source for designing wound dressings based on alginates, fucoidans, carrageenans, and ulvans, which serve as active and effective therapeutic tools. The goal of this review is to summarize available information about the modern wound dressing technologies based on seaweed-derived polysaccharides, including those successfully implemented in commercial products, with a focus on promising and innovative designs. Future perspectives for the use of marine-derived biopolymers necessitate summarizing and analyzing results of numerous experiments and clinical trial data, developing a scientifically substantiated approach to wound treatment, and suggesting relevant practical recommendations.

## 1. Introduction

The skin is one of the most important organs which, besides protecting the body from external stresses and pathogenic microorganisms, is involved in respiration, thermoregulation, and communication with the environment via receptors. Each human is always exposed to all kinds of injuries and wounds while being at home, at work, or as a result of an accident. After being inflicted, they reduce the quality of life and, therefore, any skin damage must be immediately and effectively treated. The dynamic mechanism of wound healing is a process involving a complex cascade of cellular, molecular, and biochemical responses and signaling processes triggered in a certain sequence [1,2].

The treatment of skin wounds has always been and remains a major healthcare and social issue. Every year, an immense number of people in the world get a countless number of wounds, injuries, burns, ulcers, and surgical wounds which require substantial funds and healthcare efforts for treatment. Therefore, invention of an effective wound dressing that would be affordable and easy to apply is still an urgent problem in modern medicine [1,3,4].

The history of medicine is, to a large extent, the history of the search for the most perfect wound dressing using natural materials and substances for primary medical care and specialized treatment of skin wounds [2,5,6]. In recent decades, various approaches have been developed and implemented for this purpose, including the use of special wound dressings and coatings such as polyurethane foam films, hydrocolloids, hydrogels, and paraffin dressing, which provide moisture and exudate adsorption and also delivery of active drug molecules to wound. These dressings are now increasingly demanded in the market of medical expendable supplies [1,3,4,7].

To date, the most effective treatment strategies for wound healing have been multifunctional types of wound dressings (bandages). Structurally, they include synthetic or natural biologically active substances (BAS) with mechanisms of anti-inflammatory, antimicrobial, immunostimulating, analgesic, and antioxidant action [1,5]. Modern wound bandages are designed not only for covering skin damage, but also for minimizing possible medical complications and stimulating the healing phases of various wound types. Thus, the correct choice of wound dressing type with a specific mechanism of action is crucial for the successful treatment of certain wound [4,5,8,9].

Synthetic products and materials pose a high risk of side effects: allergic complications, toxic effects, and a probability of bacterial drug-resistance [3,5,10,11]. Therefore, with the development of technologies for designing wound bandages, natural biopolymers, including those derived from marine organisms, become increasingly valuable [5,6,12].

The world’s oceans are inhabited by an abundance of organisms that differ from terrestrial ones by a significantly higher phylogenetic diversity. Due to their evolutionary adaptation to various environmental conditions, marine organisms such as algae, mollusks, sponges, and corals have acquired the ability to synthesize unique biopolymers exhibiting extremely high biological activity [1,3,13,14,15,16,17].

In the present review, we focus on marine algae, a vast community of multicellular autotrophic organisms taxonomically organized into three large groups depending on the color of their thalli: Chlorophyta (green), Rhodophyta (red), and Phaeophyceae (brown). All of them are an inexhaustible source of various polysaccharides with properties that meet the modern requirements to materials used in wound dressing designs: bioavailability, biocompatibility, non-toxicity, and lack of side effects during the process of wound healing [10,12,15].

However, the widespread use of natural polysaccharides as a structural basis for wound dressings is still hampered by a number of factors. To a significant extent, this is due to the insufficient number of successful biomedical and experimental studies on the use of marine-derived biopolymers for wound healing, as well as the lack of a systematic approach to assessing their clinical effectiveness before introduction in clinical practice [9,12,18]. Furthermore, the issue of the standardization of quality of marine-derived polysaccharides still remains unresolved. Their structural diversity depends on the season of collection, geographical location, and other factors [13,18,19,20].

The goal of the present review is to summarize available information about modern technologies for creating wound dressings based on polysaccharides derived from marine algae, including those successfully implemented in commercial products, with the focus on promising and innovative designs.

### 1.1. Wound Healing Process and Phases

A wound is a breach of the integrity of the skin, mucous membrane, internal tissues, or an organ as a result of physical, thermal damage, or trophic disturbances [5,11,12,21]. Any skin damage, whether it is a small cut or extensive and deep wound, needs care and treatment including such an important part as the application (replacement) of a bandage [11,22,23].

**Figure 1:**
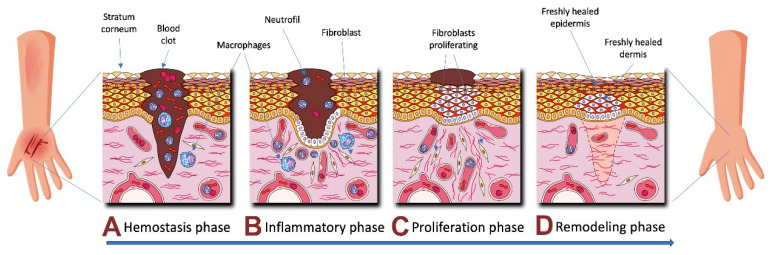
The sequence of interconnected phases of the wounds healing process: coagulation (**A**), inflammation (**B**), proliferation (**C**) and tissue remodeling (**D**).

Wound repair is one of the most complex biological processes that occur in human life. The dynamic pathogenetic process of the healing of acute wounds depends on the balance of such factors as oxygenation, temperature, and pH [12,24,25,26]. It consists of several successive and interrelated phases: coagulation (hemostasis), inflammation, fibroblast proliferation, and tissue remodeling [23,27,28,29,30] (Figure 1).

The initial stage is aimed at preventing excessive blood loss, for which it triggers the hemostatic mechanisms in response to bleeding [19,21,24]. This phase lasts for several minutes and mainly affects the epithelial and endothelial compartments, as well as blood cells. The main processes are as follows: formation of a fibrin clot and hemostasis that are provided by external and internal cascades of coagulation, cause vascular spasm, adhesion, and platelet aggregation. This releases growth factors and vasoactive molecular substances that trigger the migration of immunocompetent cells to the wound [22,31,32].

Simultaneously with the hemostasis activation, the inflammatory phase is induced (from a few minutes to 1 to 2 days, sometimes up to 2 to 3 weeks), which includes vascular and cellular responses [33,34]. Clinically, inflammation is manifested as tissue edema, infiltration, and activation of macrophages (monocytes) and neutrophils. This phase includes the sanitation of necrotic tissues, phagocytosis of bacteria, vascular responses (initial pronounced vasoconstriction followed by intense vasodilation and increased capillary permeability), and secretion of regulatory mediators that initiate the formation of granulation tissue [29,33,35,36,37].

Furthermore, the inflammatory phase is accompanied by activation of fibronectin synthesis induced by growth factors, as well as by migration of fibroblasts—pluripotent stromal cells that dominate cell populations on the first day and play a significant role in healing—towards the wound [11,30,33]. During this period, a large number of mediators are released into the wound, thus, providing the process of granulation formation, including proangiogenic growth factors that initiate the proliferation and organization of vascular endothelial cells [27,29,34,35].

The following phase, proliferation, begins at 12 h post-injury and involves a number of important processes. This period is characterized by the migration and proliferation of keratinocytes, which are the main epidermis cells, the rapid division of fibroblasts and secretion of type I and III collagen by them (up to a normal ratio of 4:1), and the formation of the extracellular matrix, which increases the strength of wound [36,37]. 

On days 10–14 post-injury, the tissue remodeling phase begins. During this phase, the synthesis and lysis of collagen comes to equilibrium, excessive macromolecules degrade, and the cellular phenotypes and the integrity of the skin restore [11,31,38,39,40]. The criterion for successful wound treatment is the epithelialization of the wound surface, which depends on angiogenesis occurring through migration, proliferation, and organization of vascular endothelial cells, as well as the functional and anatomical restoration of the skin with no visible scar mark [30,41,42,43]. 

Depending on the duration and pattern of the healing process, acute and chronic wounds are distinguished. An acute wound appears as a result of traumatic or operational (surgical) damage to the skin and heals within 8–12 weeks, depending on the size and degree of tissue injury [23,24,44,45]. Duration of the phases and sequence of the healing stages can be disturbed by a number of local and systemic factors (compression, diseases such as diabetes mellitus, hypoxia, necrosis, excessive accumulation of reactive oxygen species). A long-lasting and incomplete healing process causes chronic, recurrent, or long-term non-healing wounds (from 12 weeks or more), which often do not reach functional and anatomical recovery even after a long-term treatment [5,12,40,46].

The major objective of treatment of skin injuries is to select the proper wound dressing in order to create optimal conditions for accelerating the healing of injuries and minimizing the risk of possible complications: infection, formation of resident cell phenotypes, matrix degradation, etc. [24,31,34,47,48,49,50].

### 1.2. Classification of Wound Dressings

Since ancient times, linen and cotton bands, down, boiled wool, and woven and non-woven fabrics with various degrees of hygroscopicity, impregnated with oil, honey, resin or wine, have been used as bandage materials [1,4,6]. Their main function was to protect wounds from environmental factors, absorb wound exudate, and prevent secondary infection [43,47,51]. The discovery of the antiseptic properties of phenol, silver nitrate, zinc sulfate, wine and camphor alcohols, as well as the technique of impregnation of bandage with them, became an important milestone in wound treatment approaches in the 19th century. The subsequent advent of antibiotics, along with the invention of occlusive dressing, led to a revolution in wound treatment techniques and a sharp reduction in mortality [6,52].

Traditional sterile cotton gauze (tulle) bandages were used for many decades, until, in the 1950s, their list was extended with synthetic materials made of polymers (nylon, polyethylene, polypropylene, polyesters, etc.), which, according to modern classifications, belong to passive (inert) wound dressings [32,53,54]. In the same period, views on wound dressings radically changed: their main function was considered to be the treatment of a wound rather than simply covering it [53,54].

**Figure 2:**
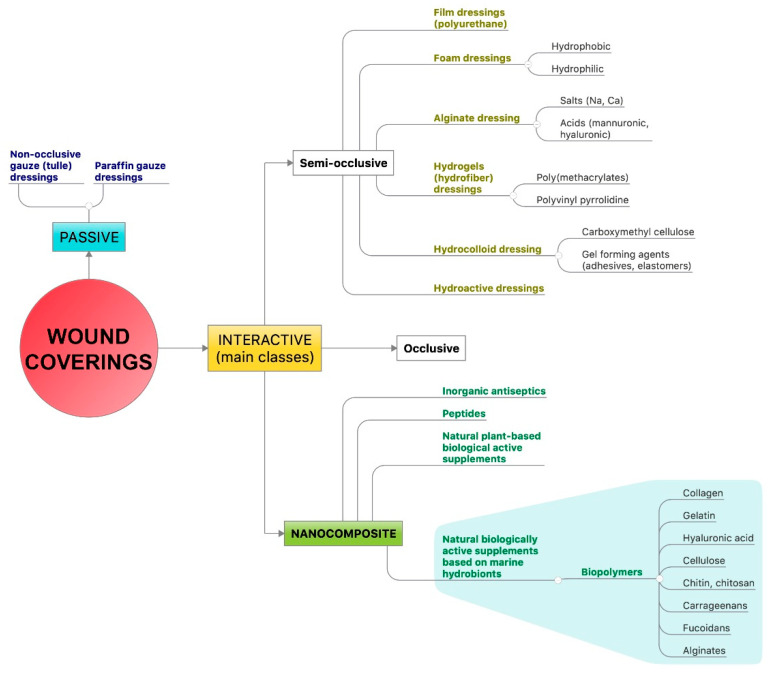
Modern classification of wound dressings is based on the origin of polymers: natural and synthetic. They are grouped into passive and interactive (semi-occlusive, occlusive and nanocomposite), including those containing natural biologically active substances.

The modern classification of wound dressings is based on the origin of polymers: natural and synthetic. They are grouped into passive and interactive (semi-occlusive, occlusive and nanocomposite), including those containing natural biologically active substances. Interactive dressings are available in the form of films, foams, hydrogels (hydro-fibers), and hydrocolloids [6]. The subject of this review is nanocomposite dressings based on marine-derived biopolymers (Figure 2).

Currently, these coatings have not lost their relevance and are used with some limitations as primary or secondary bandages for exudate removal and wound treatment. Traditional materials provide certain antibacterial protection, are passively involved in wound healing, but can damage young epithelial cells during re-bandages, and require frequent changes to prevent maceration of healthy tissues [6,53,54,55]. Among the earliest modern modifications of this type of dressing are paraffin bandages that do not injure tissues when removed. They are used for first- or second-degree burns and as skin grafts [6,39,56,57].

Until the middle of the 20th century, the generally accepted condition for successful treatment was keeping the wound dry. However, in the 1960s, the results of the research by G.D. Winter (1962) [58], and by C.D. Hinman and H. Maibach (1963) [59] were published in Nature. They showed that maintaining a moist environment in wounds provides a better effect of detritus purification without damaging cells, accelerates the formation of the vascular network, and increases the epithelialization rate by almost 50% [42,60]. The wound fluid is necessary for the normal functions of macrophages, neutrophils, keratinocytes, and fibroblasts. Furthermore, it contains proteolytic enzymes and growth factors involved in the wound healing phases [51,60,61].

To date, there are more than 500 known wound dressing types differing in composition and properties that are used in clinical practice [6,62]. The introduction of a new type of wound dressing—interactive dressings that control the microenvironment and moisture balance in the wound—in recent decades was the result of increased requirements for the treatment of skin injuries [6,52,63,64].

Modern wound dressings are required not only to be non-toxic, non-allergenic, biocompatible, biodegradable, and mechanically strong, but also to possess bioactivity: they should release included biomolecules into the wound area and play the role of a medicinal form in the healing process [52,53,65]. Furthermore, they should have a complex therapeutic effect: they should create and maintain an optimal environment for healing on the wound surface with a balanced level of moisture and for irreversible binding of wound exudate, provide gas exchange, and maintain appropriate temperature in tissues, exhibit antibacterial and antioxidant properties, stimulate cell migration, and provide non-traumatic removal after healing [6,53,66,67,68].

However, there is no single type of dressing which could be commonly used and suitable for all wounds. Moreover, in the process of wound healing, some changes occur that require wound dressings with other properties and methods of immobilization [42,68].

Since the early 21st century, there has been a steady increase in the production of commercial wound dressings that meet these requirements to a certain extent (hydrogels, hydrocolloids, sponges, films, and foams), as well as innovative dressing types. The effectiveness of modern interactive dressings (made of both natural and synthetic polymers) significantly increases when the basic structural material is impregnated with inorganic or organic antiseptics, growth factors, and biologically active substances obtained from various sources including seaweeds (nanocomposite dressings) [42,51].

Due to the clinical application of nanocomposite wound dressings, the paradigm of wound management based on the TIME concept (Tissue, Inflammation/Infection, Moisture and Edge) has emerged in recent decades. It includes wound tissue sanitation, elimination of inflammation/infection, maintenance of moisture balance, cleansing, and marginal epithelialization of the wound [65,66,67].

The modern TIME concept is a basis for the effective treatment and care of wounds. Therefore, the main characteristics of innovative bandages are associated with the development of composite (nanocomposite) structures made of natural biopolymers. These classes of wound dressings control the main healing processes by releasing active bioagents from polymer matrices into the wound: they induce thrombosis, inhibit inflammation, stimulate the migration of fibroblasts and immunocompetent cells, activate the expression of adhesion and collagen molecules, optimize regeneration, etc. [6,52,53,69,70,71,72].

In modern bandage materials, marine-, plant-, animal-, fungus-derived, or bacterial polysaccharides are used as passive natural polymer matrices. Such remarkable characteristics and unique properties as high biocompatibility, mechanical strength, flexibility, porosity, and biodegradability make natural polymers a very promising material as a framework for wound dressings [69,70,71,73,74,75,76].

A few detailed reviews considering the clinical practice and current biotechnological trends, as regards the design features, properties, mechanisms of action, and indications for the use of the main classes of wound dressings made of natural biopolymers, have been published in the dedicated Russian and world’s literature in recent years [3,4,12,31].

In accordance with the goal of the present review, we focus on modern technologies for creating polymer dressings that include biologically active compounds from marine organisms, with the emphasis on algae-derived polysaccharides and their therapeutic effect on the phases of wound healing.

### 1.3. Biologically Active Compounds from Marine Organisms

Natural products have always played a significant role in human life. They were consumed as food and applied as medicinal raw materials. For quite a long time, terrestrial plants, animals, and microorganisms were considered the main biological source of natural products [18,20]. With the active exploration of the marine environment, their list was extended to include various marine organisms which have significant taxonomic differences from terrestrial plants, animals, and microorganisms [18,60,68,76,77].

The complexity and biodiversity of marine ecosystems is associated with the extreme conditions in the world’s oceans which contribute to the synthesis of a wide and diverse range of natural substances with unique structures [15,18,20,30]. Due to their diverse biological properties, they have become a valuable and unique technological raw material for creating commercial products which are highly demanded in the biomedicine market, not only as a matrix, but also as therapeutic components of wound dressings [2,5,76].

In the last 60–70 years, thousands of biopolymers with unique chemical structures were isolated and characterized from marine organisms. Most of them proved to be superior to the known natural substances obtained from terrestrial organisms in their biological and pharmacological activities [2,14,15,16,17,18,44,45,53,76,78]. This explains the increased interest of physicians, biologists, chemists, and biotechnologists in the study of marine metabolites with potent anti-inflammatory, analgesic, antioxidant, antibacterial, and procoagulant activities to be used for medical purposes [14,20,60,61]. This interest is associated with their unprecedented biotechnological potential for the development of modern nanocomposite wound dressings [14,55,59,68].

In recent decades, a significant number of experimental and clinical studies have been conducted to clarify the ability of marine biopolymers (alginates, ulvans, chitosan, chitin, carrageenans, fucoidans, etc.) to modulate certain phases of the wound healing process [2,16,19,20,30,44,45,46,48]. Their significant potential to influence the focus of injury by inhibiting inflammation, activating fibroblast proliferation, and remodeling tissues has been revealed. This influence is mediated by a variety of associated mechanisms that have a synergistic action on the overall effectiveness of local treatment, which proved to be especially pronounced in the case of using polysaccharides from seaweeds [2,19,53,55,59,79,80,81].

## 2. Polysaccharides from Marine Algae Used in the Development of Wound Dressings

Marine algae are among the most ancient inhabitants of the planet. These are photosynthetic organisms with complex and peculiar taxonomy [18,19,20,42]. Currently, two main types of algae are distinguished: microalgae, consisting of a single eukaryotic cell, are widely represented in marine ecosystems as phytoplankton, and macroalgae, having large sizes, are a heterogeneous group occupying the intertidal zone [55,56,59,70].

Over millions of years of existence in the marine ecosystem, they have developed effective mechanisms of antibiotic protection against pathogenic microorganisms and numerous strategies of survival under extreme abiotic conditions of the environment [2,10,21,44,45,46,47,48,49]. During evolution, these organisms acquired the ability to synthesize a wide range of metabolites and biomolecules, many of which have a unique chemical structure that other organisms do not have [10,53,54,55,56,76,77]. Polysaccharides from marine algae are of particular interest due to the high resistance, biological activity, and availability of these organisms in large numbers [21,59,61,69,74].

The cultivation of marine macroalgae with subsequent extraction of polysaccharides and their use for a variety of purposes, including therapeutic, due to their antiviral, antibacterial, immunomodulatory, and antitumor activities, has been a major focus of interest since long ago [19,55,59,61].

The unique healing properties of algae have been known and used in wound treatment for many centuries. For these properties, sailors called them “mariner’s cures” [79]. Over the past few decades, rich experience has been accumulated in the use of numerous homo- and heteropolysaccharides, widely represented in the main classes of marine algae, as a therapeutic basis for wound dressings [14,15,16,17,47]. In recent years, these biopolymers (consisting of monosaccharides linked via glycosidic bonds) have attracted increasing attention as biotechnological raw materials for pharmacology, food, and cosmetic additives [14,15].

The increased technological opportunities for the isolation and purification of these polysaccharides have substantially expanded the range of their practical and potential applications as a basis for various types of wound dressings [2,48,49,53,56,75]. Due to their chemical and physical characteristics, such as mechanical strength, emulsification, adhesive properties, the ability to form hydrocolloids, and non-toxicity, they have a more pronounced healing effect compared to that shown by their traditional natural counterparts [49,53,56].

In recent decades, hydrogels, which are three-dimensional hydrophilic polymer chains consisting of 99% water, have become an example of the widespread use of seaweed-derived polysaccharides in wound dressing design and tissue engineering [70,74]. Due to the high biocompatibility, low immunogenicity and cytotoxicity, as well as the ease of functioning, these polymer systems are now actively used in wound treatment [10,21,44,54,69,70,71,72].

The 3D reticulate structure of hydrogels simulates the microarchitectonics of extracellular matrix of native tissue, acts as a physical barrier against bacteria, providing optimal conditions in vivo for cell survival [21,45,55,61]. Furthermore, the attractiveness of seaweed-derived polysaccharides to be used as a material for creating hydrogels is explained by their biological activity, biocompatibility and biodegradability, as well as by the opportunity of physical and chemical modification of their structure [2,10,69,76,77].

The structural attractiveness of this type of wound dressings is enhanced by including nanofillers with antimicrobial and anti-inflammatory activities (gold, silver, zinc and copper oxides, antibiotics, hormones, etc.) in their composition [2,21,44,55,69].

Biocompatibility and biodegradability are particularly attractive characteristics of seaweed-derived polysaccharides, due to which they can simulate the extracellular matrix to a certain extent [2,48,75]. Such properties have raised significant biotechnological interest in these biopolymers used as a therapeutic basis to design bandage materials for already several decades [2,46,47,55,56].

In particular, a significant therapeutic potential was revealed in classes of various polysaccharides, the major components that perform important structural functions in algae (fucoidans, alginates, carrageenans, ulvans, cellulose, and laminarins) [10,21,48]. Their content in algae has seasonal and species-specific variations, and their constant trait—high hydrophilicity—fully fits the modern concept of creating moist conditions for wound treatment [48,49]. This feature of seaweed-derived polysaccharides makes them immediate agents in the wound healing process and an indispensable natural biomaterial for designing various types of modern wound dressings [48,74,77].

Below is a more detailed consideration of the best-known polysaccharides that are already widely applied or promising components of wound dressings with proven effectiveness and high potential for wound healing.

### 2.1. Alginates

Alginates, being polysaccharides derived from the class of brown algae (genus *Fucus*), are considered among the world’s most common marine biopolymers. They have long been effectively used as a gelling agent and stabilizer of various solutions and suspensions, as well as a valuable component in food, chemical, and biotechnological productions. These polysaccharides are an indispensable component of various products manufactured in the pharmaceutical and medical industry [77,79,80]. The unique characteristics of these metabolites have found application as a therapeutic basis for nanocomposite wound dressings [78,80,81].

**Figure 3:**
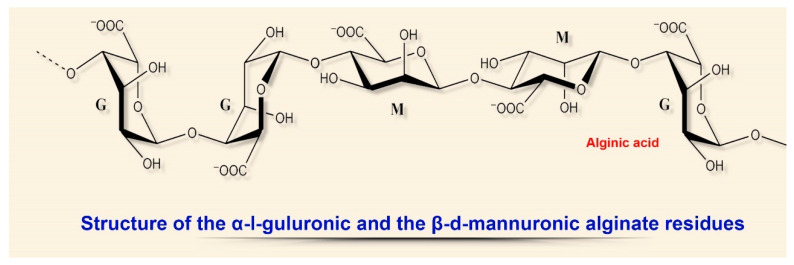
Structural diversity of algal sulfated polysaccharides: α-I-guluronic and β-d-mannuronic alginate residues.

The physical and chemical properties of these linear acidic polysaccharides depend on the structural ratio of the two types of uronic acids, L-guluronic (G) and D-mannuronic (M), located in the biomolecule in the form of homo- or heteropolymer blocks (Figure 3).

The most common technology for obtaining hydrogels from an aqueous alginate solution is the combination with an ionic crosslinking agent that is divalent cations (e.g., Ca^2+^, Ba^2+^, or Co^2+^) interacting with G-fragments of polymer chains [80]. Calcium alginate wound dressings with a high content of G-blocks have a lower rate of ion (Ca–Na) exchange with exudate, slowly swell, but are atraumatic and painless when removed [82,83].

Analogous dressings with a high content of M-blocks quickly absorb wound exudate, but require special irrigation when removed [82,83,84]. The ability of sodium ions to form transverse bonds with alginate makes these porous dressings an almost perfect barrier membrane for tissue engineering and targeted tissue regeneration [23,25,29,85].

In recent reviews, the structural features and chemical properties of alginates and variants of their use in modern medicine were considered more in detail [25,86].

When modern interactive nanocomposite wound dressings are created, the most valuable biological and pharmacological characteristics of these natural polyelectrolyte biopolymers include the biocompatibility, non-toxicity, biodegradability, as well as high hemostatic activity associated with the release of calcium ions which activate platelets and other clotting factors [79,80,87,88]. The potent procoagulant properties of these anionic biopolymers were proposed to be used in the composition of calcium-sodium gel dressings for healing various types of wounds as long ago as in the second half of the 20th century (Kaltostat^TM^, Kaltocarb^TM^, Kaltoclud^TM^) [79].

At the same time, it was found that alginates included in wound dressings, in addition to the high hemostatic activity, provide the optimum moist environment in the wound and good absorption of wound exudate (20-fold relative to the dressing weight), stimulate the growth of granulation tissue, reduce the concentration of pro-inflammatory cytokines, inhibit the formation of free radicals, and have a pronounced antimicrobial activity [80,87,89]. Clinically, this is manifested as a reduction in the healing time and longer intervals between bandagings, which also become painless and atraumatic [80,87,88,90].

Due to their other, but no less important biotechnological properties (such as low cost, availability, and high biocompatibility), alginates are widely used in modern commercial hydrogel wound dressings in combination with metal ions for the treatment of acute and chronic wounds: diabetic ulcers, bedsores, and traumatic and surgical wounds (Algicell^TM^, AlgiSite^TM^ M, Comfeel^TM^ Plus, etc.) [88,91,92,93,94].

Furthermore, film and foam dressings based on sodium alginate also seem to be very promising. These types of wound dressings improve wound healing by normalizing gas exchange, protecting wounds from infection, especially in combination with other biopolymers, essential oils, or surfactants that enhance dispersion [89,92,93].

The modern world pharmaceutical market offers a great variety of different types of alginate-based bandages, from traditional hydrogel dressings to innovative lyophilized sheets and nanofibers for cavity wounds [27,46,95,96,97], as well as combined designs of these polysaccharides with Zn, Mn, Ag, glycerol, polyvinyl alcohol, and other marine-derived polymers [94,98,99,100].

For example, K. Murakami with co-authors [87] managed to effectively implement the wound healing properties of alginates in combination with other marine BAS (fucoidan, chitin/chitosan) and mitomycin C in a design of hydrogel-based wound dressing [87]. The results of experimental studies show that this combination of marine biopolymers has a large number of properties of perfect dressing for wound healing: chemoattractant effect on fibroblasts, activation of their proliferation, as well as acceleration of tissue re-epithelialization and granulation, which began to appear on day 7.

In the analysis of the mechanisms of healing action of this wound dressing, attention is drawn, first, to the effective combined action of brown algae polysaccharides, alginates and fucoidans, whose low mechanical strength in this case is compensated by chitin and chitosan [95,101,102].

Recently, polysaccharide-containing hydrogel bifunctional platforms based on a combination of alginate and hyaluronic acid, which are successfully used in cosmetology [23,29,80,101,102], with hyaluronan, its derivative, have shown themselves well in wound healing. As it turned out, hyaluronan decelerates the release of Ca^2+^ ions, regulates the alginate gelation, and, at the initial stages of healing, provides moisture for the wound, activates migration and proliferation of keratinocytes [85,101,102,103,104].

Moreover, with the overall biological efficiency of alginate-containing hydrogel monoplatforms, the gelation process is a difficult-to-control stage, which results in heterogeneity of the gel structure and its unsatisfactory mechanical strength [101,105]. In an experimental model, gel-like mixtures based on the alginate–hyaluronic acid combination showed a faster wound healing effect due to a positive influence on the gelation kinetics [101,102,106]. In addition, alginate–hyaluronan hydrogel structures exhibit the potential to be a platform for delivering biologically active compounds directly into the wound [102,107].

Thus, alginates have long and firmly been recognized as a therapeutic basis for numerous and diverse designs of modern commercial wound dressings. The high biocompatibility, sorption properties, and ease of gelation have provided the widest popularity of these biopolymers in biomedical science, biotechnology, and tissue engineering (Table 1).

However, the production of raw materials from marine brown algae for modern biotechnologies and the creation of wound dressings is not limited to the use of these polymers.

### 2.2. Fucoidans

**Figure 4:**
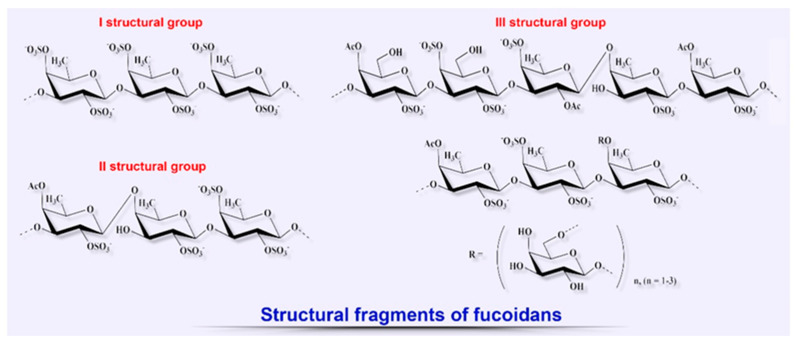
Structural diversity of algal sulfated polysaccharides: fragments of fucoidans.

Since the late 20th century, the number of scientific studies aimed at elucidating the therapeutic potential of other biopolymers from brown algae such as fucoidans for the treatment of various diseases, including the wide range of their biological properties to be used for wound healing, has shown a tendency to increase (Figure 4) [76,87,120,121,122,123,124,125].

It has been established that this class of anionic sulfated heteropolysaccharides is present only in brown algae. Some marine invertebrates (sea urchins, Japanese sea cucumber) can synthesize similar polysaccharides [15,17]. Their structure is composed only of sulfated fucose residues and is regular, which significantly distinguishes them from fucoidans [16,17]. The chemical composition, structure, and biological properties of fucoidans are strongly dependent on the environmental conditions, season of collection, species of algae, and also on the technologies used for their fractional extraction and purification [69,74,121,122,124].

Different proportions of the structural monosaccharides constituting fucoidans such as fucose (the main monomer), glucose, galactose (which is also sometimes the main monomer), xylose, mannose, as well as sulfate ester and uronic acid, determine the pattern of biological activities of these biopolymers [69,87,125]. There are widely known commercial make-up products based on highly purified fucoidan extract (Maritech^®^ Reverse and Vita-Bright™), which exhibits pronounced regenerating, protective, and anti-aging properties for skin. However, the ability of fucans to modulate certain phases of wound healing by activating biomolecules and cellular processes has attracted the significant interest of biotechnologists in recent decades [122,123,124].

For example, the presence and position of sulfate groups are important factors that determine the anti-inflammatory properties of these biopolymers, including the inhibitory activity of cell proliferation, peroxidation, and neutrophil migration, and also their properties as agents of the cell–receptor interaction and potent anticoagulants [69,122,124,125]. In terms of the mechanism of anticoagulant action, low-molecular-weight fucoidans resemble heparin; they are a potent inducer of the production of such multifunctional cytokine as hepatocyte growth factor/scatter factor (HGF/SF), which plays an important role in the process of wound healing and re-epithelialization, stimulation of angiogenesis, and migration and proliferation of keratinocytes [126,127].

As R. O’Leary with co-authors showed in their research [128], besides the above-mentioned HGF/SF, some varieties of these polysaccharides derived from brown algae of the genus *Fucus* actively interact with the transforming growth factor TGF-β, which is a potent cytokine regulating cell proliferation, differentiation, apoptosis, immune response, and remodeling of the extracellular matrix [128]. In an experimental model of acute wounds caused by punch biopsy, the level of the TGF-β factor increased rapidly, which caused scars to form at the healing site. Fucoidans inhibited the antiproliferative effect of TGF-β, significantly increased the rate of repopulation of wound by fibroblasts and the rate of formation of the fibrillar collagen matrix, thus, being promoters of wound healing.

M. Kordjazi with co-authors [69] were among the first to study the wound-healing effects of fucoidans in a burn wound experiment. The researchers paid attention to the anticoagulant, antithrombotic, anti-inflammatory, and antioxidant properties of these polysaccharides, whose activity depended on the degree of sulfation (from 32.6 to 19.0%). With a higher sulfate content, the wound-healing properties of fucoidans were more pronounced, which was manifested as the degree of activation of fibroblast proliferation (which is recognized as the main mechanism), collagen deposition, and an increase in the epidermis thickness.

Similar studies, conducted later, confirmed these results. A conclusion was made that low-molecular-weight fucans containing increased levels of sulfates and fucose accelerate skin wound healing through a complex and coordinated antioxidant, anti-inflammatory, and growth-dependent activity [20,120,124,129].

The high potential of using fucoidans as the basis to create wound dressings was shown in the research of Australian scientists who used fucoidan extracted from the alga *Fucus vesiculosus* to design a special polyelectrolyte multilayer assembly in combination with chitosan. According to the authors, the results obtained can contribute to the invention of promising dressing materials [125].

In their recent studies, J. Cashman and A. Charboneau with co-authors revealed a high potential of fucoidans to inhibit the formation of post-operative adhesions in abdominal wounds [129,130]. The authors used film-based wound dressings and preparations containing fucoidans derived from *F. vesiculosus* in experiments with rabbit and rat models.

The potential and the identified properties of these polysaccharides are especially demanded in view of the high frequency (65–95%) of adhesions formed after surgical operations in the abdominal and pelvic regions according to medical reports [63]. Therefore, the prevention and treatment of adhesion process is a public healthcare issue. Unfortunately, the search for barrier methods preventing adhesions has not been successful for many years [63,129].

Nevertheless, the pathogenetic process of adhesion formation is quite well studied. The main mechanism of the adhesive process was found to be associated with the adhesion activation through the local inflammatory process mediated by surgical damage to the peritoneum, and the subsequent exudation of plasma rich in fibrin into the cavity [63,129].

The major, fundamental conclusion made by the authors based on the results of the experiments is that, among the numerous substances tested, fucoidans proved to be the most effective anti-adhesive, non-toxic agents and were considered as a promising candidate for clinical use [129,131]. Therefore, implementation of the high potential of these polysaccharides in the form of hydrogels, films, and solutions for the prevention of adhesive process is highly probable in the coming years [129,130].

It should be recognized that, despite the experimentally proven wide range of biological activities of fucoidans, the practical application of the wound healing properties of these polysaccharides in the form of wound dressings is still under development [132,133]. To date, none of the fucoidan-based products has received official approval for clinical use, despite years of efforts to purify them and study their biological activity [131,134]. However, the results obtained give hope that it may possibly happen in the coming years.

### 2.3. Carrageenans

**Figure 5:**
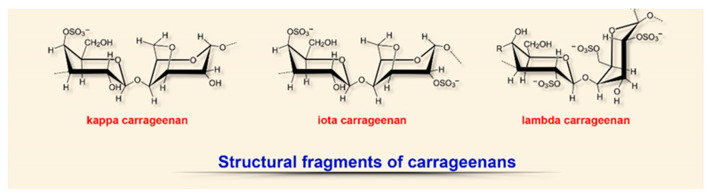
Structural diversity of algal sulfated polysaccharides: fragments of carrageenans.

Over the past few decades, the activity of studies on structurally diverse metabolites from red marine algae (Rhodophyta) having useful biological properties has substantially increased [20,135,136,137]. These algae contain chlorophylls, carotenoids, and xanthophylls, as well as pigments specific for this group: phycoerythrin and phycocyanin. The characteristic color and the name of algae are related to the presence of these pigments in different quantitative proportions (Figure 5) [135,137,138].

Carrageenans, a group of high-molecular-weight sulfated polysaccharides obtained from marine algae of the division Rhodophyta, attract particular attention as a rich and renewable source of phycocolloid polysaccharides [18,135]. Currently, these structural components of algae membranes are considered as a promising source of biopolymers with a unique structure and specific physical and chemical properties [20,136,137,138].

The structure of these anionic sulfated polysaccharides (polygalactans) consists of alternating linear chains of α-1,3-galactose and β-1,4,3,6-anhydrogalactose with ester sulfates (15–40%) and resembles natural glycosoaminoglycans [18,20,135,136,137]. Depending on the degree of sulfation (from 15 to 40%), solubility and source of extraction, six types of carrageenans are distinguished, of which ϰ (kappa), ι (iota), and λ (lambda) are the most fully characterized and studied [135,136,137]. The viscoelastic and gelling properties of these polysaccharides, as well as the presence of many functional groups in the structure (hydroxyl and sulfate), make these biopolymers a perfect material as a gelling agent in the design of hydrogel-based wound dressings with various chemical modifications [138,139]. 

A few noteworthy reviews that focus on the transformation of characteristics of carrageenans depending on changes in their structure and chemical and physical properties have been published in recent years [Zhang, Shankar, Zia, Cunha]. Therefore, based on the goals of the present review, we here consider only the main trends in the use of these common and promising polysaccharides for wound healing as a basis for the design of various wound dressing types.

Among various polysaccharides from red algae, ϰ-carrageenan has certainly been studied best of all for the purpose of the development of hydrogel-based wound dressings (as the most common type of wound dressing). In addition to biocompatibility, this biopolymer type exhibits pronounced hemostasiological and immunomodulatory properties necessary for healing [135,140,141].

Hydrogels are formed as a result of heat-reversible gelation, ion cross-linking, or photo-cross-linking of methacrylate modifications of the backbone of this biopolymer [137,138]. In contrast to the simpler ionic cross-linking of polysaccharide in the presence of K^+^ or Ca^2+^, leading to the formation of brittle hydrogels [138,142], the incorporation of methacrylate groups of photo-cross-linking in the main ϰ-carrageenan backbone, followed by activation with UV irradiation in the presence of a chemical photoinitiator, provided greater stability of reticulate gel [142,143].

Gradient hydrogels based on ϰ-carrageenan and gelatin exhibit noteworthy healing properties and, therefore, have many advantages compared to conventional layered or reticulated analogues [142]. The gradual and smooth variation in one of the physical properties of the material (viscosity, porosity, or density) simulates the tissue environment in vivo and has a positive effect on cell morphology [138,142,143].

Promising types of ϰ-carrageenan-based hydrogels are nanogels that structurally contain medicinal nanoparticles of up to 100 nm and release them at a rate dependent on the temperature in the wound (37–45 °C), as well as hydrogels created by 3D-bioprinting with the desired shape and specified mechanical properties and chemical structure [142,143,144,145]. These forms of carrageenan-based hydrogels are excellent excipients for the prolonged release of not only antimicrobial agents, but also bioactive molecules and growth factors [143].

For example, in their recent study, H. Li with co-authors [144] developed a promising strategy for three-dimensional bioprinting of a multilayered structure with strong interphase bonds using cationic (gelatin) and anionic (ϰ-carrageenan) hydrogels [144]. The proposed structure was strong and also stable at 37 °C, providing high viability of cells in the wound.

The various antiviral and antibacterial activities of carrageenans, as well as their anti-inflammatory and immunomodulatory properties revealed in recent years, have raised additional interest in them from the biotechnological and pharmaceutical aspect as wound-healing biodressings [135,136]. The insufficient mechanical strength of these polysaccharides is compensated for by the addition of various natural or synthetic polymers: polyvinylpyrrolidone, polyethylene oxide, polyvinyl alcohol, hyaluronic acid, or locust bean gum [137,146,147,148].

For example, in a recent experimental work, A.V. Nair with co-authors [149] studied the wound-healing properties of β-(1–3) (1–6) glucan/carrageenan hydrogels. The presence of carrageenan in the composition increased the porosity of gels and activated the attachment and proliferation of fibroblasts in experiments in vivo and in vitro, with a more rapid wound healing as compared to the control [149].

Intact skin is known to have slightly acidic pH values (4.0–6.0); with bacterial infection, skin pH increases to an alkaline level (up to 9.0). Colonization of wounds by pathogenic bacteria negatively affects treatment, and, therefore, controlling pH as a biomarker of infection is important for assessment and monitoring of healing [150].

K. Zepon with co-authors [137] reported, for the first time, the development of a combined “smart” wound dressing: a pH-sensitive hydrogel film based on covalent binding of ϰ-carrageenan polysaccharide, locust bean (*Ceratonia siliqua*) gum, and cranberry extract [137]. In this design, locust bean gum enhanced the mechanical properties of carrageenan hydrogel. Another component of the coating, the anthocyanin-rich cranberry extract, is not only an antibacterial agent, but also acts as a sensitive pH indicator which changes its color in the case of an alkaline reaction in the wound fluid, thus indicating bacterial infection.

Thus, due to their almost perfect physical and chemical properties, carrageenans have found a wide range of applications as a basis for designing wound dressings. The presence of several functional groups in the composition, the high hydrophilicity and the strong negative charge of these polysaccharides allow the modification of their properties and enhancement of their biological activity in a wide range.

### 2.4. Ulvans

**Figure 6:**
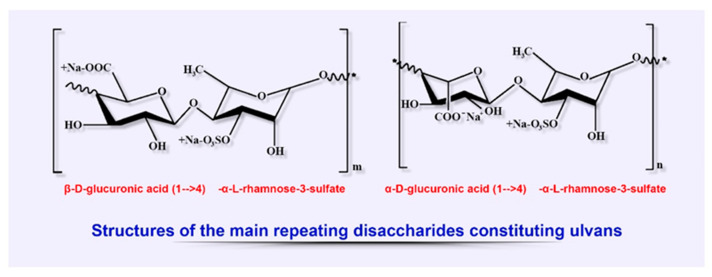
Structural diversity of algal sulfated polysaccharides: main repeating disaccharides constituting ulvans.

Ulvans, classified as a group of sulfated heteropolysaccharides, are among the main biopolymers extracted from cell wall of some members of green algae, the class Ulvales (species of Ulva, Enteromorpha, and Utricularia). Ulvans, being a component of the cell wall, provide osmotic stability and cell protection along with other polysaccharides of these algae (cellulose, xyloglucan, and glucoronan), constituting up to 45% of dry weight (Figure 6). [20,151,152].

The chemical composition of ulvans strongly depends on the species of algae, the season of their collection, the habitat conditions during growth, and extraction methods. The typical structure of these polyanion heteropolysaccharides is represented by rhamnose, xylose, glucose, galactose, uronic acids (glucuronic and iduronic), as well as by sulfate and carboxyl groups structured as the main ulvanobiuronic (aldobiuronic) acid disaccharides designated as glucurorhamnose 3-sulfate (types A) and iduronorhamnose 3-sulfate (type B) [18,153].

Ulvans are almost insoluble in organic solvents, which is explained by the relative hydrophobicity of rhamnose [20,153,154,155,156]. This property limits the opportunities of chemical modifications of ulvans and prevents their potentially wide application in wound dressing design [151,156,157]. However, in solutions with high pH, the conformation of these polysaccharides increases the intermolecular interactions in the wound, which makes it possible to obtain hydrogels with high viscosity [152,154,155]. This feature allows the transformation of the gel-forming properties of polysaccharide by manipulating the structural and functional relationships [152,153].

The presence of charged sulfate and carboxyl groups in the structure complicates obtaining mechanically stable hydrogels, which is associated with the active water absorption and development of hydrolytic degradation [151,154,156]. When wound dressings are designed, these structural features of ulvans necessitate, on the one hand, solving the problem of their preliminary modification to make them insoluble and, on the other hand, increasing the mechanical properties of gels [154,156]. The latter problem is solved by creating compound ionotropic gel complexes with cationic polymers or inorganic additives such as boric acid, copper, calcium, zinc, or magnesium [154,158].

The presence of rare carbohydrates, iduronic acid, and sulfated rhamnose in the biochemical profile of ulvans is a feature distinguishing them from other seaweed-derived polysaccharides [155,159]. Thus, the presence of rhamnose enhances the biological activity of ulvans, especially in the treatment of skin pathologies (by influencing the biosynthetic pathways in dermis), and also improves wound-healing properties (by reducing bacterial adhesion and stimulating cell proliferation and collagen biosynthesis) [152,159,160,161,162].

The primary structure of these polysaccharides is directly related to the wide range of their macromolecular properties that determine the pharmacological attractiveness of ulvans and their potential to be used in biomedicine. Experimental and model-based studies have revealed significant antioxidant [13,154,155], anticoagulant [15,153,154], antitumor [17,152,156,157], antihyperlipidemic [13,17,152,154], and immunomodulatory [13,16,153,159] biological activities of ulvans, both in vitro and in vivo. Moreover, ulvans, like all seaweed-derived sulfated polysaccharides, exhibit a wide range of antiviral activities. These biological features of ulvans have found application not only in the treatment of a number of diseases as a preventive anti-biofilm agent, but also in the design of bandages for wound treatment and tissue engineering [152,155,157,161,162].

An example of successful application of the physical and chemical properties of this polysaccharide is the ulvan-chitosan polyion complex gel developed by K. Kanno with co-authors, which proved to be more stable than an alginic acid-chitosan gel both in acidic and basic conditions. However, under model conditions, this complex was inferior to the heparin-chitosan gel-coated vessel in terms of anticoagulant properties [153].

Thus, studies on the biological properties of ulvans and their biotechnological potential for creating wound dressings are only at an initial stage, as compared to studies on other seaweed-derived polysaccharides. The structural features of these complex biopolymers require more attention to elucidate their effect on the wound process phases and subsequently propose substantiated recommendations on their use in specific types of wound dressings.

## 3. Conclusions

Seaweed-derived sulfated polysaccharides are a new and promising biologically active source for creating wound dressings. The significant structural diversity and presence of various functional groups provide their high potential for managing the wound healing process and stimulating the mechanisms of natural skin regeneration. The hydrophilic property of these marine polysaccharides determines their ability to form hydrogels that can absorb exudate from wounds and create a moist environment necessary for successful healing.

Various types of wound dressings based on alginates, fucoidans, carrageenans, and ulvans act as active and effective therapeutic tools. They are involved in wound healing not only as natural dermis simulators, but also as functional biomaterials for controlled drug delivery, cell immobilization, and tissue bioengineering technologies.

Being easily cultivated and nonfastidious, marine algae have become an inexhaustible natural resource of valuable polysaccharides with unique biological properties. Over the past few decades, they have turned into an attractive alternative to synthetic dressings.

The experience of experimental studies on the feasibility of pathophysiological regulation of wound healing phases using sulfated polysaccharides has been accumulated for years of research, in parallel with the extension of our knowledge about the pathogenetic mechanisms of wound processes. This required summarizing the data of clinical studies and analyzing the effectiveness of various types of wound dressings with marine biopolymers based on the objective results obtained.

The abundance of attractive and promising technologies for the creation of natural dressings based on sulfated polysaccharides necessitates finding a scientifically grounded approach to wound treatment and proposing relevant practical recommendations. This will provide wider opportunities for their application in the treatment of wounds of various etiologies.

The future prospects for the use of seaweed-derived sulfated polysaccharides largely depend on the interaction between clinicians and biotechnologists engaged in the development and testing of modern dressings. Nevertheless, the search for novel wound dressings based on seaweed-derived polysaccharides, as well as for the forms of their application, is far from being completed.

## Figures and Tables

**Table 1:** Examples of commercially available alginate wound dressings based on seaweed polysaccharides.

Commercial Name	Feature	Benefits	Refs
AlgiCell^®^ Ag (Integra LifeSciences Corp.)	Antimicrobial gel high-strength calcium-alginate dressing with complex silver ion transfer technology (1.4%)	- Consists of a patented mixture of D-mannuronic and L-guluronic acids, which provides good gelation and high moisture resistance. - High absorption capacity - Removing the dressing does not leave any residue of silver coated nylon thread in the wound	[23,29,81,108,109]
Derma AlgiCell^®^ (Integra LifeSciences Corp.)	This is a soft, sterile calcium alginate dressing. Consists of a mixture of D-mannuronic and L-guluronic acids.	- absorbs from moderate to a large amount of exudate. Covers or fills the wound cavity. - Easily and painlessly removed during dressings. - Maintains a moist wound environment.	[86,89,108]
AlgiCell^®^ (Integra LifeSciences Corp.)	Nonwoven wound dressings, dressings, or fibers.	Upon contact with exudate, these dressings form a wet gel during ion exchange. - High gel strength, painless and atraumatic removal from the wound. - Well maintains moisture in the wound.	[94,99,108]
AlgiSite^®^ M (Smith and Nephew, Inc.)	An alginate dressing containing calcium forms a hydrophilic gel upon contact with exudate.	- Helps prevent scab formation and helps reduce wounds. Easy painless removal when changing. - Optimizes gas exchange in the wound bed.	[82,94,106,110]
Amerx^®^ (Amerx Health Care Corp.)	Dressings in the form of a sterile, elastic pad containing calcium.	- High absorbency, quickly forms a hydrophilic gel to create and maintain optimal moisture in the wound. Convenient packaging, easy to use.	[84,111]
Biatain^®^ Alginate (Coloplast Corp.)	High-performance alginate dressings with high absorbent properties.	- Available in waterproof format. - It has hemostatic properties. - High biocompatibility. - Optimal drainage of wound exudate.	[80,93,112]
Cuticerin™ Gauze Dressings (Smith and Nephew, Inc.)	Alginate mesh dressing soaked in neutral hydrophobic euserin ointment, petroleum jelly, paraffin.	- Impregnated soft acetate fibers reduce the risk of granulation tissue growing through the dressing.	[20,94,108]
CarboFLEX^®^ (ConvaTec)	Hydrocolloid, sterile, non-adhesive, five-layer dressing for direct contact with the wound, absorption of odors.	- Specially designed to solve control problems associated with unpleasant odors on the wound.	[20,88]
Carbonet^®^ (ConvaTec)	A multi-layered, flexible and soft odor-absorbing dressing that is highly adaptable to wound contours	- Specially designed to solve control problems associated with unpleasant odors on the wound. - Forms a soft, hydrophilic, gas-permeable gel upon contact with exudate.	[20,42,80,108]
CovaWound™ (Covalon Technologies, Ltd.)	A primary wound dressing made from the calcium salt of alginic acid rich in D-mannuronic acid.	- The dressing follows the contours of the wound and provides a microenvironment that promotes wound healing.	[18,41,42,113]
Cutimed^®^ Alginate (Essity)	Hydrogel alginate dressing, has a high absorbency and helps maintain a moist environment in the wound.	- Maintains a moist environment in the wound - Fast gelation upon contact with exudate. High gel stability. Highly absorbent base provides effective drainage of the wound.	[20,22,42,108]
DermaGinate™ 12” Rote (DermaRite Industries, LLC)	Calcium-alginate dressing that easily fills the wound bed.	- Forms a calming gel-like consistency upon contact with wound exudate. - Easily and painlessly removed during dressings.	[20,41,92]
DermaGinate/Ag™ (DermaRite Industries, LLC)	Silver-alginate dressing. It limits the growth of bacteria in a dressing to reduce the risk of secondary infection of wounds.	- Effectively sorb from moderate to significant volume of exudate. Easily fills a bed of wounds. - Creates a soothing gel-like consistency upon contact with exudate in the wound. - Maintains moisture in wounds.	[42,92]
DynaGinate™ (DermaRite Industries, LLC)	A sterile dressing made of calcium alginate, designed to protect the wound and maintain its moist environment.	- It has a high absorption capacity, which is designed to absorb moisture 17 times its own weight. - Easily forms a gel in contact with exudate, maintains wound moisture and speeds up the healing process.	[42,92]
ExcelGinate™ (MPM Medical, Inc., USA)	Primary non-woven calcium alginate dressing for partial or full thickness of wounds with moderate or severe drainage.	- Tightly woven, when removed, the integrity of the coating is fully preserved. - Highly absorbent coating properties, absorbs four times its weight. - Can be used on infected wounds.	[20,42,82,114]
Fibracol™Plus (Systagenix)	Combination dressing of 90% collagen and 10% alginate.	- Maintains integrity when wet. - Does not stick when removed, does not leave fibers in the wound. - Alginate helps maintain a moist environment in the wound, stimulates the formation of granulation tissue and epithelization.	[20,42,83,115]
GEMCORE360° ™ (GEMCO Medical, USA)	Reinforced dressing with calcium alginate in the form of antimicrobial foam soaked in polyhexamethylene biguanide	- Maintains a moist wound environment. - Forms a soft, flexible hydrophilic layer of gel upon contact with exudate. - Active against a wide range of bacteria (including MRSA, MRSE, VRE, *Escherichia coli, Klebsiella pneumoniae, Pseudomonas aeruginosa*, *Candida albicans* и *Rhodotorula mucilaginosa*)	[20,84,116]
Kalginate™ Thin (DeRoyal, USA)	Alginate primary dressing of heavy fibers for adsorption of exudate.	- Absorb exudate up to 20 times its weight. - Great for daily dressings. - Forms a soluble sodium gel upon contact with liquid contents in the wound. - Available in coating or turunda options	[49,85,117]
KALTOSTAT^®^ Alginate Dressing (ConvaTec, UK)	The alginate dressing forms an absorbent gel-fiber matrix in contact with the liquid.	- Supports a moist wound environment and facilitates atraumatic removal. - Used for infected wounds under the supervision of a medical professional.	[21,42,79]
KALTOSTAT^®^ Alginate Rope (ConvaTec, UK)	Forms an absorbing matrix of gel fiber in contact with wound fluid	- Can be used for tamponade with nosebleeds.	[21,42,79]
3M™ Tegaderm™ High Integrity (3M + KCI, USA)	Highly resistant alginate coating containing adsorbent	- Provides highly stable gelation and optimal wet environment. Compatible with 3M dressings. - Increased absorbent properties of the dressing - High comfort for the patient during dressings.	[80,118,119]
3M™ Tegaderm™ High Integrity (3M + KCI, USA)	Provides good gelation	- Forms a mechanically strong gel, plugging the entire cavity of the wound. - High comfort for the patient during dressings. - Compatible with 3M dressings.	[80,118,119]
